# Novel *Doublesex* Duplication Associated with Sexually Dimorphic Development of Dogface Butterfly Wings

**DOI:** 10.1093/molbev/msab228

**Published:** 2021-07-29

**Authors:** Fernando Rodriguez-Caro, Jennifer Fenner, Shivam Bhardwaj, Jared Cole, Caleb Benson, Alexandra M Colombara, Riccardo Papa, Matthew W Brown, Arnaud Martin, Ryan C Range, Brian A Counterman

**Affiliations:** 1 Division of Biological Sciences, University of Montana, Missoula, MT, USA; 2 Department of Biological Sciences, Auburn University, Auburn, AL, USA; 3 Department of Integrative Biology, University of Texas, Austin, Austin, TX, USA; 4 Department of Biological Sciences, University of Puerto Rico—Rio Piedras, San Juan, PR, USA; 5 Department of Biological Sciences, Mississippi State University, Mississippi State, MS, USA; 6 Department of Biological Sciences, The George Washington University, Washington, DC, USA

**Keywords:** wing pattern, Lepidoptera, gene expression, ultraviolet, sexual dimorphism

## Abstract

Sexually dimorphic development is responsible for some of the most remarkable phenotypic variation found in nature. Alternative splicing of the transcription factor gene *doublesex* (*dsx*) is a highly conserved developmental switch controlling the expression of sex-specific pathways. Here, we leverage sex-specific differences in butterfly wing color pattern to characterize the genetic basis of sexually dimorphic development. We use RNA-seq, immunolocalization, and motif binding site analysis to test specific predictions about the role of *dsx* in the development of structurally based ultraviolet (UV) wing patterns in *Zerene cesonia* (Southern Dogface). Unexpectedly, we discover a novel duplication of *dsx* that shows a sex-specific burst of expression associated with the sexually dimorphic UV coloration. The derived copy consists of a single exon that encodes a DNA binding but no protein-binding domain and has experienced rapid amino-acid divergence. We propose the novel *dsx* paralog may suppress UV scale differentiation in females, which is supported by an excess of Dsx-binding sites at cytoskeletal and chitin-related genes with sex-biased expression. These findings illustrate the molecular flexibility of the *dsx* gene in mediating the differentiation of secondary sexual characteristics.

## Introduction

Explaining the origin of morphological variation is a major challenge in evolutionary and developmental biology. Some of the most dramatic intraspecific variation in nature emerges from sexual dimorphism such as the striking colorations of birds of paradise, the antlers of male elks, and elaborate horns of rhinoceros beetles that result from the evolution of sex-specific developmental pathways (Sibley 1957; Jarman 1983; Emlen et al. 2007; Coyne et al. 2008). Such traits often evolve under the influence of sexual selection (Emlen 2001; Emlen et al. 2007; Irestedt et al. 2009) and are key elements of the evolutionary ecology of species. The development of these sexually dimorphic traits in males and females that have nearly identical genomes is often achieved through patterns of sex-specific gene expression.

Sexually dimorphic development involves the doublesex/mab-3 related (*Dmrt*) family of transcription factors that share a common DNA-binding domain (Kopp 2012). *Dmrt* genes are responsible for initiating cascades of tissue-specific gene expression patterns that drive populations of cells to develop with male or female fates. In insects, the *doublesex* gene (*dsx*, also known as *Dmrt1*) functions as a molecular switch at the base of the sex determination cascade through alternative splicing, a highly conserved mechanism across all orders of insects studied (Price et al. 2015; Hopkins and Kopp 2021). Interestingly, *dsx* has been independently co-opted multiple times during the evolution of novel secondary sexual dimorphisms (Kunte et al. 2014; Deshmukh et al. 2020). For example, in *Drosophila*, tissue-specific expression of alternative *dsx* isoforms is essential for proper development of a myriad of sexually dimorphic traits (Verhulst and van de Zande 2015). In butterflies, *dsx* underlies the repeated evolution of female-limited mimetic wing patterns (Nishikawa et al. 2015; Iijima et al. 2019; Palmer and Kronforst 2020) and is required for the development of male-specific scent organs (Prakash and Monteiro 2020). These findings highlight the importance of *dsx* in the evolution of insects, not only as an ancient and highly conserved developmental switch, but also as a ubiquitous element in the evolution of novel sexually dimorphic traits (Burtis and Baker 1989; Kimura et al. 2008; Kijimoto et al. 2012; Ito et al. 2013).

**Fig. 1.**
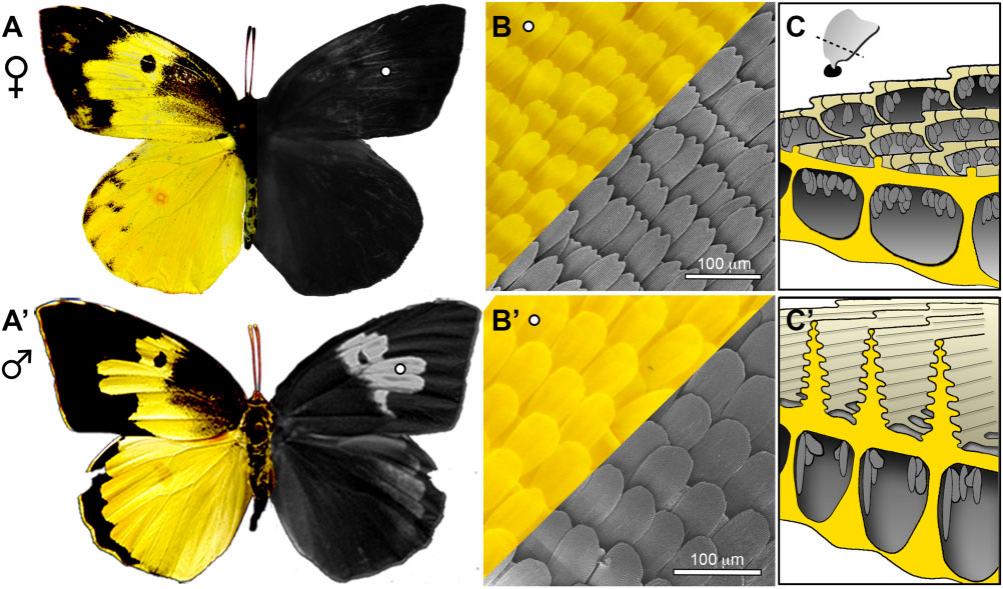
Sexual dimorphic UV patterns on *Zerene cesonia* wings. (*A*–*A′*) Female and male visual (left) and UV (right) color patterns. (*B*–*B′*) Light and scanning electron microscope (SEM) images show differences in cell arrangements in females and males (images taken from wing region denoted by white circle in *A*). (*C*–*C′*). Drawing renditions of scale cross-sections highlighting differences in chitin cytoskeletal differences between female non-UV reflecting scales and male UV-reflecting scales (based on focused ion beam SEM images from Fenner et al. [2019]).

Coliadinae butterflies provide an opportunity to test the ubiquity of *dsx* in the evolution of novel phenotypes. In this group, sexually dimorphic ultraviolet (UV) wing patterns have evolved rapidly, resulting in dramatic diversity within and between species (Silberglied and Taylor 1973; Rutowski 1977; Kemp and Rutowski 2011; Stella et al. 2018; Pecháček et al. 2019). Studies across the Coliadinae clade have found that UV dimorphism is frequently a sexually selected trait involved in speciation (Kemp et al. 2005; Kemp 2008). Therefore, UV reflectance has been a highly influential trait in the diversification of Coliadinae butterflies. In the *Colias* genus, UV brightness has been shown to be the greatest indicator of male mating success and important for interspecific mate choice (Rutowski 1977; Silberglied and Taylor 1978; Papke et al. 2007). In the sister genus, *Zerene*, UV patterns also differ between species and likely influence mate choice and reproductive isolation when they come into contact and hybridize in nature (Gerould 1914). Here, our objective is to examine the potential role of *dsx* and sex-specific gene expression in the development of UV-reflective patterns on wings of the Southern Dogface Butterfly, *Zerene cesonia* (Lepidoptera: Pieridae) (Fenner et al. 2019). This trait is male limited in *Z. cesonia*, where elaborate structures on the scales in narrow regions of fore and hind wings reflect a bright UV pattern that resembles the profile of a “dog’s face” (fig. 1). Although the UV-reflective patterns vary dramatically between the sexes of *Z. cesonia*, the pigmentation patterns are largely identical, allowing us to leverage differences in sex-specific expression patterns to explore the genetic basis of novel UV reflecting structures that have evolved in a subset of pierid butterflies (Wilts et al. 2011). Using RNA-seq and immunolocalization, we characterize patterns of sex-specific gene expression through male and female wing development, with a particular focus on 24–72 h APF when scales visibly develop and differentiate (Ghiradella 1989; Dinwiddie et al. 2014). This study provides a detailed examination of the genes involved in sexually dimorphic wing development, reveals a novel duplication and mode of sex-specific expression of *dsx*, and highlights the importance of *dsx* in the evolution of novel phenotypes.

## Results

### Sex-Biased Expression during Wing Development

**Fig. 2.**
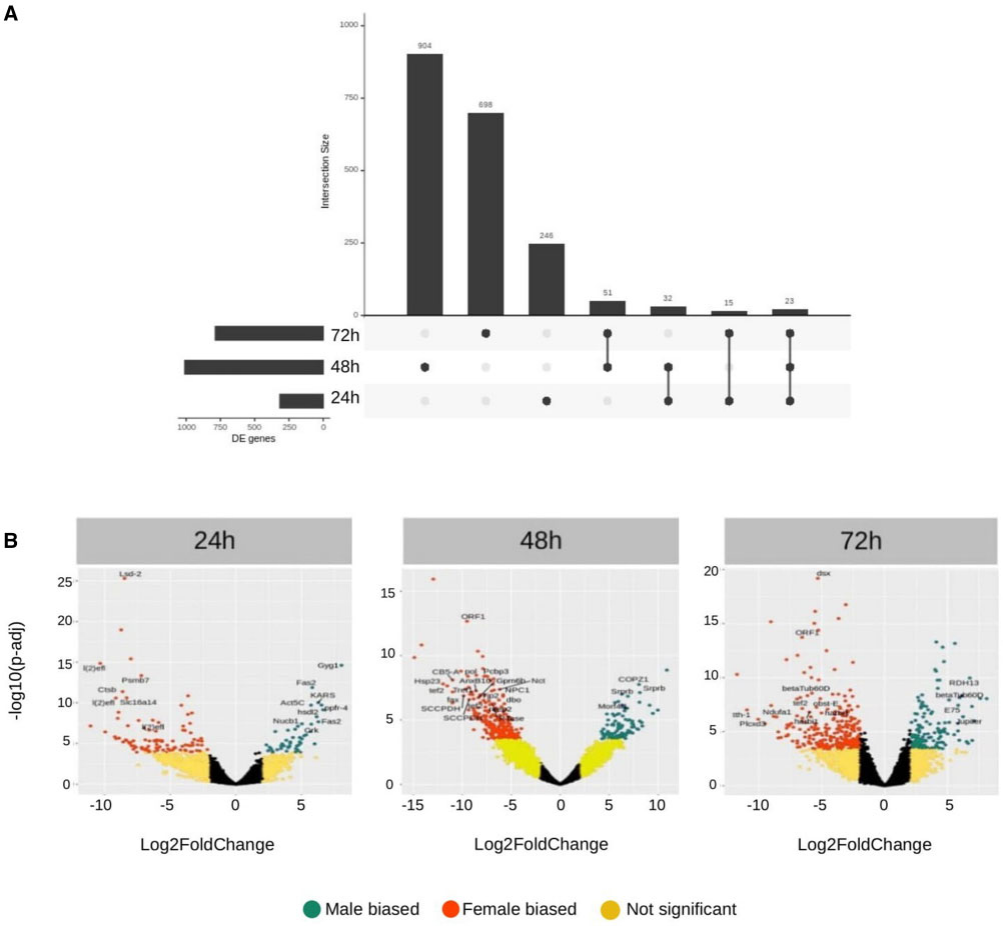
Sexually dimorphic gene expression in *Zerene cesonia* wings. (*A*) UpSet plot showing overlap between three sets of DE genes (24, 48, and 72 h APF). The horizontal bars show the number of DE genes at each stage, with 48 h AFP exhibiting the greatest number of DE genes. Vertical bars show the number of unique DE genes for each stage and set of stages. (*B*) Volcano plots of DE genes at each developmental stage from 24 to 72 h showing the distribution of female and male-biased genes. Note that *dsx* is the most DE gene at 72 h AFP.

We identified 2,584 transcripts differentially expressed (DE) in the interval from 24 to 72 h after pupal formation (APF) (*P*-adj < 0.05, | log2FC | > 2) with a peak of differential expression at 48 h APF and little overlap of DE transcripts between stages (fig. 2*A*). Of these, 774 showed male-biased expression and 1,810 female-biased expression (fig. 2*B*). The distribution of sex-biased genes often varies between autosomes and sex chromosomes (Ellegren and Parsch 2007). In Lepidoptera, the Z chromosome often has an excess of genes with male-biased expression (Huylmans et al. 2017). However, in *Z. cesonia* wing development the numbers of male-biased and female-biased genes on the Z chromosome were not significantly different from expectations based on random distributions (Fisher exact test *P* values of 0.72 for females and 0.31 for males). Despite there being no excess of male-biased genes on the Z chromosome, the average expression of Z-linked genes was significantly higher in males compared with females (Wilcoxon test, *P* = 0.03), indicating that dosage compensation is not complete. When compared with autosomal genes, the median expression level of Z-linked genes is 21% lower in males (Z:A ratio = 0.79) and 27% lower in females (Z:A ratio = 0.73). These results suggest that in *Z. cesonia* wing development, the Z chromosome exhibits incomplete dosage compensation, with overall lower dosage from the Z chromosome compared with autosomes in both sexes but higher expression of Z-linked genes in males relative to females.

### Novel *Dsx* Duplication Shows Sexually Dimorphic Expression

*Dsx* had the most sexually dimorphic expression of all the DE genes during wing disc development (log_*2*_FC = 5.321621, *P*-adj = 3.2e-16). Unique *dsx^F^ and dsx^M^* transcripts were expressed in female and male wings, respectively; similar to what has been previously reported in *Drosophila and Bombyx*. De novo transcriptome assembly of individual RNA-seq libraries resulted in *dsx^F^* transcripts for every female and *dsx^M^* transcripts for every male (supplementary fig. S1, Supplementary Material online). Unexpectedly, the *dsx^F^ and dsx^M^* transcripts mapped to two distinct copies of *dsx* in the *Z. cesonia* genome assembly (supplementary fig. S2, Supplementary Material online), suggesting that instead of alternative splicing, *dsx^F^ and dsx^M^* originate from the expression of paralog sequences.

**Fig. 3.**
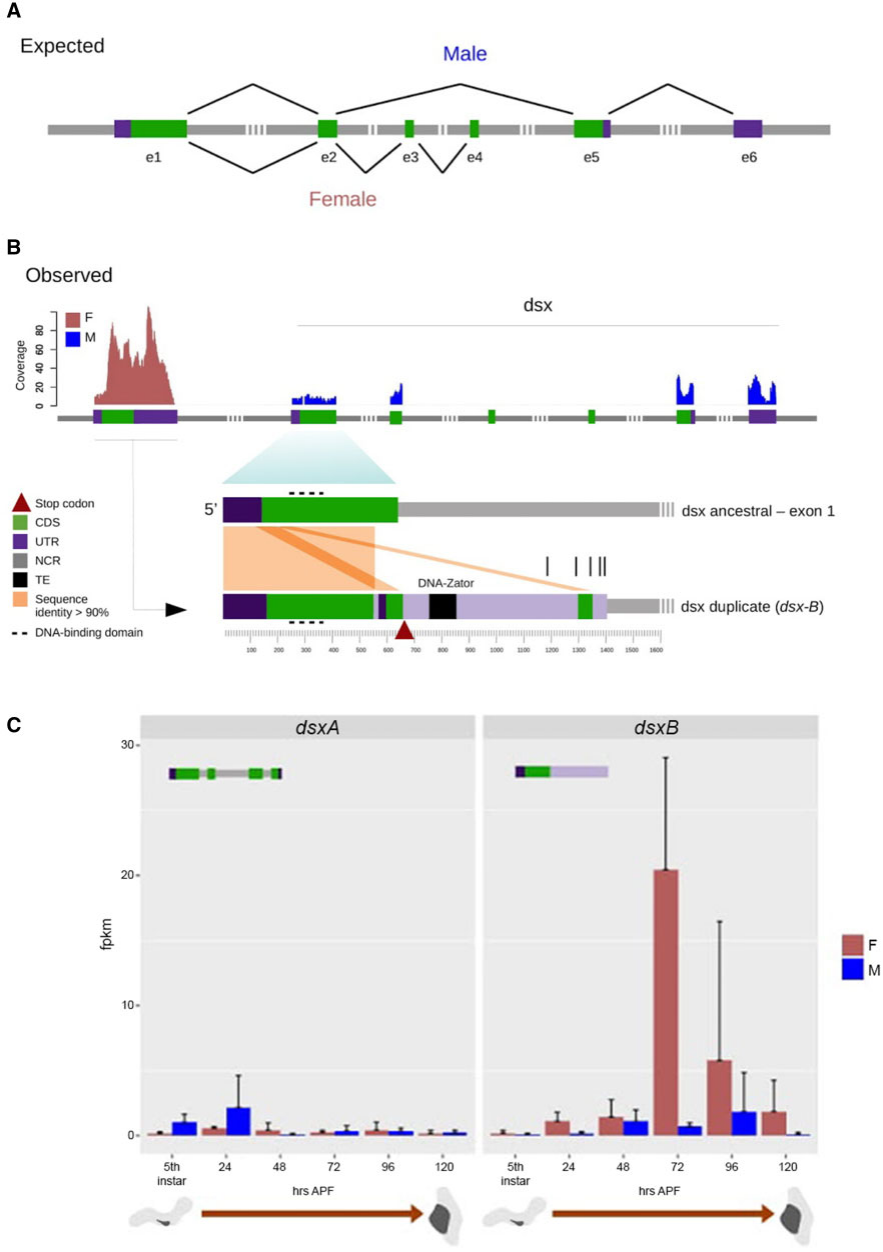
*Dsx* duplication and expression in *Zerene cesonia*. (*A*) Splicing patterns of *dsx^F^ and dsx^M^*, observed in most arthropods. (*B*) Observed *dsx* duplication and gene annotations. An incomplete tandem duplication of the *dsx* gene was found in the genome of *Z. cesonia*. The barplots on top show read coverage of *dsx* paralogs from a male (blue) and a female (red) sample, after removing multimapping reads. The bottom diagram shows a sequence alignment of *dsx* paralogs and a detailed annotation of the novel paralog (*dsxB*) including the predicted UTRs, stop codon and coding region based on the longest open-reading frame. In this diagram, vertical bars on top of *dsxB* indicate the different lengths of independently assembled *dsxB* transcripts from single-library assemblies. (*C*) Quantification of *dsxA and dsxB* expression through wing development from RNA-seq data. Error bars show 95% confidence intervals calculated from biological replicates.

In the *Z. cesonia* genome assembly, there are two tandem annotated copies of *dsx* on chromosome 8 (fig. 3*A*). Duplications of *dsx* have never been reported in a Lepidoptera species, thus we first sought to annotate and confirm the duplication was not an artifact of the genome assembly. A full-length *dsx* gene (denoted *dsxA*) sequence was annotated that spanned ∼10 kb and included the six exons and UTR sequences found in *dsx* genes of other Lepidoptera as well as *Drosophila* (Baral et al. 2019) (fig. 3*B*). A partial duplicate of *dsx* (denoted *dsxB*) was also found that includes multiple partial duplications of the 5′ UTR and exon 1. The first ∼535 bp of *dsxB* contains a near complete duplication of the *dsxA* 5′ UTR and CDS of exon 1 with >90% sequence similarity (fig. 3*B* andSupplementary fig. S3, Supplementary Material online). This region is followed by ∼30 bp of unknown origin or sequence similarity with *dsxA*, and then ∼100 bp that includes a second duplication of the 5′ UTR and exon 1, again with >90% sequence similarity to *dsxA*. The next ∼550 bp show no sequence similarity to exons or introns of *dsxA* and include a ∼100-bp novel transposable element (TE) insertion (DNA Zator Pieris 5178). Following the TE insertion, there is another ∼50 bp of the duplicated exon 1 that again is >90% similar to *dsxA* and appears to have been split from the nearest upstream duplicated segment of exon 1, likely due to the TE insertion. Collectively, this annotation suggests *dsxB* is the result of multiple duplication events and a recent TE insertion.

The *dsx* duplication appears to have occurred recently in the lineage leading to *Zerene* butterflies, followed by a relatively rapid accumulation of amino acid substitutions. We were able to successfully amplify and sequence *dsxB* from the sister species *Zerene eurydice*, confirming the duplication occurred before the species split. However, we were unable to confirm the presence of a *dsx* duplicate in the sister genus *Colias* using sequence similarity searches in the *Colias eurytheme* or *Colias croceus* genome assemblies. Attempts to amplify *dsx* in *C. eurytheme* using primers from conserved sequence regions and *dsxB*-specific primers from *Z. cesonia* also failed to amplify more than one *dsx* copy. Phylogenetic analyses of the *dsx* sequences from *Zerene* sps. (*dsxA and dsxB*), *Colias eurytheme* (*dsxA*), and *Pieris rapae* (*dsxA*), support that the duplication event likely occurred relatively recently (∼10.8 Ma) after the split with *Colias* (∼25.85 Ma), but before the split of *Z. cesonia* and *Z. eurydice* (*∼*1.76 Ma), the only two species in the *Zerene* genus (supplementary fig. S6, Supplementary Material online). The *dsxB* sequences in *Zerene* show a relatively high number of amino acid substitutions from *dsxA* sequences compared with copies of *dsxA* in *Z. cesonia, Z. eurydice*, or *Colias eurytheme*, which show no amino acid differences between species (supplementary table S1, Supplementary Material online). Correspondingly, the ratio of substitution rates at nonsynonymous and synonymous sites (*d*N/*d*S) are 10-fold higher or more between paralogs within *Zerene* species (0.2–0.209), than for *dsxA* sequences between species (0.001–0.019), further supporting rapid divergence of the novel *dsx* paralog.

The sequence divergence between the *dsx* paralogs allowed RNA sequence reads to be unambiguously mapped to *dsx* copies and revealed sexually dimorphic expression of the *dsx* paralogs during wing development (supplementary figs. S1–S3, Supplementary Material online). Nearly all RNA-seq reads from females mapped to *dsxB*, whereas the male reads primarily mapped to the *dsxA* paralog (fig. 3*B and C*). In addition, among the de novo RNA-seq assemblies every *dsx* transcript assembled from a male (*dsx^M^*) aligned best to *dsxA* and every *dsx* transcript from a female (*dsx^F^*) aligned best to *dsxB* (supplementary fig. S2, Supplementary Material online). Figure 3*B* shows reads mapped for a single male and female to the *dsxA and dsxB* copies, respectively, illustrating the sex-specific expression of the *dsx* paralogs.

Female *dsxB* transcripts often extended beyond the stop codon at nucleotide position 660 and included 3′ noncoding sequence (fig. 3*B*). These transcripts include the DM domain characteristic of *dmrt* genes (Raymond et al. 1998) including the DNA-binding domain (fig. 3*B*), a nuclear localization signal (NLS), and oligomerization domain 1 (OD1) (Verhulst and van de Zande 2015). However, for *dsxB* transcripts, we were unable to find evidence of oligomerization domain 2 (OD2) that is involved in cofactor recruitment (Verhulst and van de Zande 2015). This is not surprising, as OD2 is typically formed through sex-specific alternative splicing of *dsx* exons 3–5 in other insects, but these exons are missing in the *Z. cesonia dsxB* paralog (fig. 3).

In contrast to findings in the wing, other tissues showed evidence of sex-specific splicing of *dsxA*. cDNA sequences from adult and late larval genitalia showed evidence of male- and female-specific isoforms of *dsxA*, but no evidence of *dsxB* expression (supplementary fig. S4, Supplementary Material online). In all male tissues examined (pupal wings, adult head, adult thorax, adult, and larval testes), a *dsx* isoform was found that included *dsxA* exons 1, 2, 5, and 6 (supplementary fig. S4, Supplementary Material online). However, in female tissues (adult head, adult thorax, adult, and larval ovaries), the *dsx* isoforms included *dsxA* exons 1–6 and showed no evidence of exon skipping (supplementary fig. S4, Supplementary Material online). Interestingly, of all tissues examined in males and females, we only found *dsxB* sequences in developing female wings.

### Novel *Dsx* Paralog Involved in Female-Specific Pattern Development

**Fig. 4.**
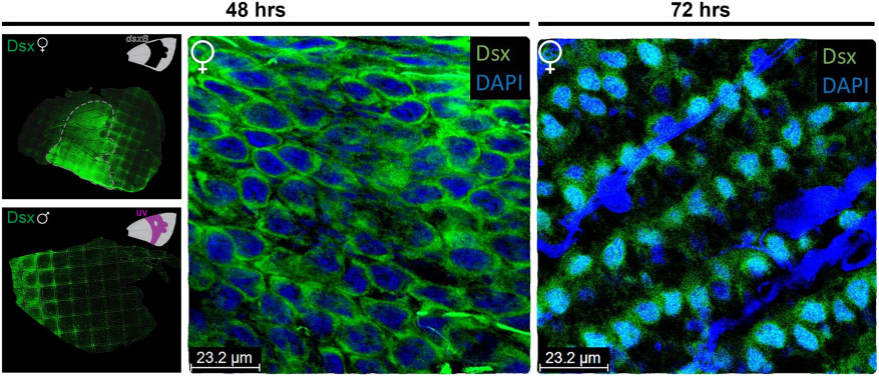
Dsx spatial expression during *Zerene cesonia* wing development. Dsx immunofluorescent stains in female (upper) and male (lower) forewings at 48 h APF. Female panel shows Dsx associated with the central pattern region of the wing (dotted white line), where UV-reflecting scales develop in males (shown in wing schematic in upper corner). Higher magnification shows Dsx in cytosol at 48 h APF (middle panel) in female ground and cover scales. At 72 h APF (right panel), Dsx is detected in the nucleus of alternating rows of scales on female wings.

The novel *dsx* paralog (*dsxB*) expression in females appears to be associated with wing color pattern elements. Immunostaining for Dsx in developing pupal wings revealed a strong signal across the central region of the wing that corresponds to the region of yellow scales responsible for generating the so-called “dogface” profile (fig. 4). This result is consistent with the RNAseq data that suggest a burst of *dsxB* upregulation in 72 h APF female wings (fig. 2*B*). In contrast, males did not show expression of Dsx in this central wing region where the UV-reflecting scales are located. Because in-situ hybridization (supplementary fig. S7, Supplementary Material online) and RNA-seq data showed evidence of *dsxA* in male and not female wings, we can infer that these antibody stains in females reflect the spatial expression of *dsxB*. Therefore, we suggest that the novel *dsx* paralog, *dsxB*, has an expression pattern associated with the development of yellow, non-UV-reflecting scales in the central pattern of female wings.

A closer look at the scale cells of female wings revealed a coordinated change of *dsxB* expression in the cytosol and nucleus between 48 and 72 h APF (fig. 4). At 48 h APF on female wings, Dsx appeared to be almost exclusively in the cytosol in ground and cover scales. However, at 72 h APF Dsx was diffusely present in both the cytosol and the nuclei. Notable is that at 72 h Dsx was present in alternating rows of scales, suggesting it is expressed in only ground or cover scales, but not both, which contrasts with 48 h APF where rows of both ground and cover scales showed *dsxB* (fig. 4). These expression patterns are intriguing, but leaves us with new questions, such as what is the possible function of the sharp differences of Dsx in the cytosol and nucleus from 48 to 72 h? Is Dsx in the female scale nuclei at 72 h APF the result of active transport from the cytosol to nucleus? A nuclear localization sequence (NLS) is present in the *dsxB* paralog, suggesting transport is possible. Although the impact of these Dsx spatial and temporal patterns remains an enigma, the timing of the changes and spatial coordination among alternating rows of different scale types (i.e., ground vs. cover) is indicative of *dsxB* playing an important signaling role in scale differentiation.

### Sex-Biased Expression Enriched for Genes Involved in Cytoskeletal Structure

**Fig. 5.**
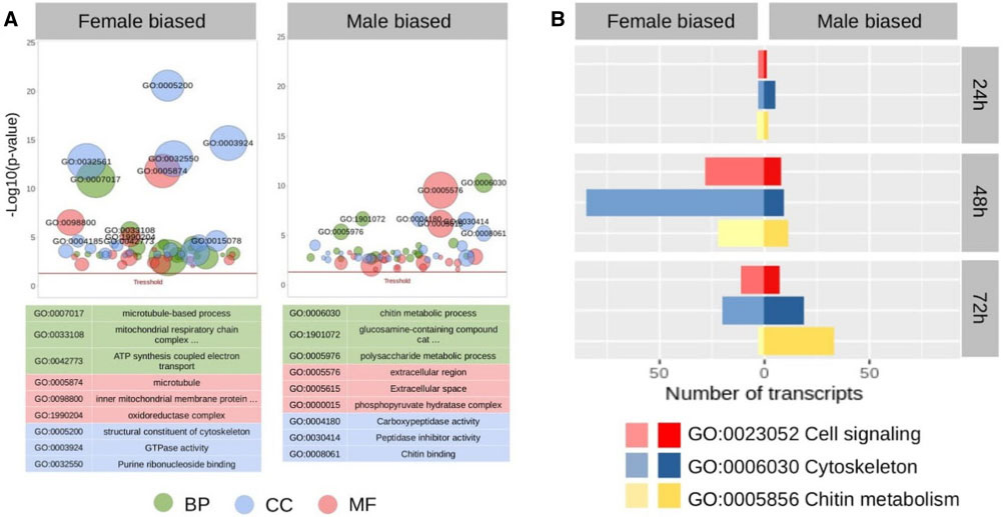
Sexually dimorphic gene expression in *Zerene cesonia* wings. (*A*) Bubble graph results of GO enrichment analyses for female and male-biased genes from 24 to 72 h APF (red line denotes *P* > 0.05); BP = Biological process, CC = Cellular component, MF = Molecular function. (*B*) Comparison of transcriptome activity related to cell signaling, chitin metabolism, and cytoskeleton development among males and females. Bars reflect the number of annotated transcripts expressed at each developmental stage (FPKM > 1).

At 48–72 h APF, morphological differences in scale structure become clearly visible (Ghiradella 1989; Fenner et al. 2019). Tests for enrichment of Gene Ontology (GO) terms among the DE genes between males and females from 48 to 72 h APF identified several biological functions related to cytoskeletal development, chitin metabolism, and cell signaling (fig. 5). In female-biased genes, an excess of these processes was most pronounced at 48 h (fig. 5*B*) and included several microtubule (MT)-related genes (fig. 5*B*). This contrasted with the male-biased genes that did not show an excess of MT-related genes, but rather showed an excess of genes involved in chitin metabolism and cytoskeletal-related processes (fig. 5). The excess of these genes was greatest at 72 h APF (fig. 5*B*), which corresponds to when UV-reflecting scale structures become visible during wing development.

**Fig. 6.**
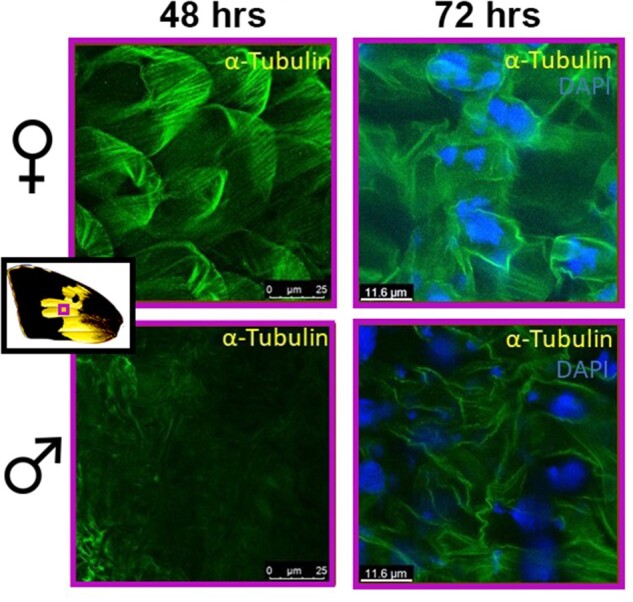
MT distributions on male and female wings (48 and 72 h APF). MTs show greater density in female scales and MTs are largely absent in developing UV-reflecting scales of males at 48 h APF.

To determine if these sex differences in GO results reflect visible differences during scale development, we stained for MTs in developing male and female wings (fig. 6 and supplementary fig. S7, Supplementary Material online). As predicted, there were clear, large differences in α-tubulin expression between males (*n* = 5) and females (*n* = 5) at 48 h APF (fig. 6). Most notable was the lack of α-tubulin in the UV reflective region of male wings. In accordance with GO enrichment results, females showed high densities of α-tubulin across the wing (fig. 6 and supplementary fig. S8, Supplementary Material online). As wing development progressed, there was little difference in MTs among males and females, and by 120 h APF, MT organization clearly reflected differences in scale structure between yellow and black scales in both sexes (supplementary fig. S8, Supplementary Material online). Together these results suggest the differential expression between males and females was enriched for genes involved in the sexually dimorphic development of the UV-reflecting scale structures.

### Male-Biased Genes Are Enriched for *Dsx*-Binding Motif

To test if *dsxB* could be acting as an activator and/or repressor of sex-specific gene expression, we used a position weight matrix of *Drosophila dsx*-binding motifs (Clough et al. 2014) to test for *dsx*-binding sites in promoter regions (2 kb 5′ and 3′ of the coding sequence) of female (*n* = 497) and male-biased genes (*n* = 289) at 72 h APF. At 72 h APF, *dsxA and dsxB* showed little or no evidence of expression in males, however, this is when *dsxB* is most highly expressed in females. Interestingly, we found a significant enrichment of *dsx*-binding motifs among the male-biased genes (Fisher’s exact test, *P* = 0.0219), but not the female-biased genes (Fisher’s exact test, *P* = 0.356) (supplementary fig. S9, Supplementary Material online). These results suggest *dsxB* may be repressing genes in females that are highly expressed in males (i.e., male-biased genes).

Unfortunately, attempts to study the functions of *dsx* in male and female wing development using CRISPR-based editing were not informative, as none of >450 injected individuals survived beyond third larval instar, preventing the examination of wing development. A 1.7-fold increase in hatching was observed by reducing the injected gRNA by half, suggesting *dsx* edits impact survival, thereby limiting the applicability of CRISPR mosaic knock-out methods to study *dsx* function. Similarly, a recent study in *Bicyclus* butterflies found a relatively low success rate in using CRISPR to edit and study *dsx* function in developing wings (Prakash and Monteiro 2020).

## Discussion

### A *Dsx* Repressor Model of UV Scale Development

**Fig. 7.**
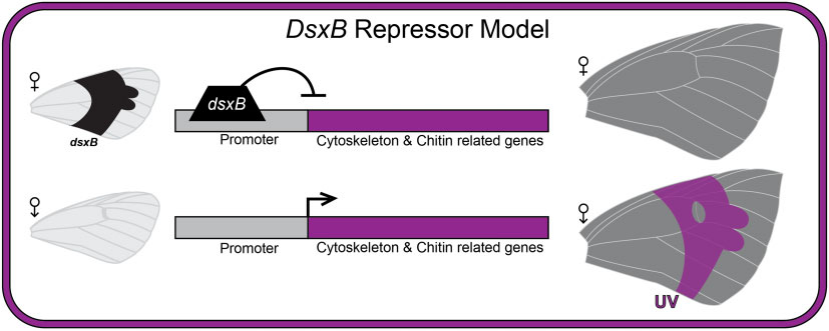
Hypothetical repressor model of *dsxB.* Model shows *dsxB* binding to promoter regions and repressing expression of cytoskeleton and chitin related genes in female pupal wing development. Wings on the left illustrate *dsxB* spatial expression during pupal wing development. Wings on the right show UV patterns in adult wings. Based on this model, *dsxB* expression in females represses the development of UV-reflecting scales, and the lack of *dsxB* in males results in the development of UV-reflecting scales.

We propose a genetic model for the regulation of UV pattern that involves the novel *dsx* paralog, *dsxB*, acting as a repressor of genes required for the development of UV-reflecting scales (fig. 7). At 72 h APF, *dsxB* is highly expressed and present in the nucleus of scales in females, but not males (figs. 2–4). Also at 72 h APF, there is a significant excess of cytoskeletal and chitin-related genes (*n* = 52) that are expressed in males and downregulated in females (fig. 5*B*). We suggest these are candidate genes involved in the development of the chitinous male-specific UV-reflecting scale structures that occur at 72 h APF (Supplementary table S2, Supplementary Material online).

We found that these male-biased genes at 72 h APF also show a significant enrichment of *dsx* binding sites in their promoter regions. Therefore, it is plausible that *dsxB* binds these promoter regions and represses these genes’ expression in females at 72 h APF. If *dsxB* can act as a repressor, we may also predict that the female-biased genes at 72 h APF should not be enriched for *dsx*-binding sites, or *dsxB* could have repressed their expression. Accordingly, there was no significant enrichment of *dsx*-binding motifs in the female-biased genes at 72 h APF. The lack of a OD2 protein-binding domain in *dsxB* also supports that it more likely functions as a repressor, than activator of transcription. Without a protein-binding domain to recruit, it is unlikely for *dsxB* to act as an activator that binds other components of the transcription factor complex (i.e., cofactors). In our model, it is unlikely that *dsxB* is repressing transcription by competing with or blocking *dsxA* function, as *dsxA* shows no or little expression in females and males at 72 h APF. Instead, promoter-bound *dsxB* may interfere with regulating the battery of effector genes that mediate the differentiation of male-specific features, including the peculiar UV-reflective scales. Further experiments that assay *dsxB* binding or function, such as CHiP-seq and CRISPR-based gene editing are needed to confirm how *dsxB* and the male-biased genes are involved in UV scale development.

Interestingly, *dsx* genes in several ant and termite species also lack the protein-binding domain (Jia et al 2018; Miyazaki et al. 2021). In several ant species, sex-specific alternative splicing of *dsx* occurs as in other insects, despite losing the OD2 protein-binding domain. There appears to be sex- and caste-specific exon usages of ant *dsx* genes, without the OD2 domain. There is also evidence of rapid, positive selection on *dsx* genes in ant lineages that lost the OD2 domain. In these ants, the sex-specific *dsx* isoforms lacking OD2 may have evolved ways to regulate sexually dimorphic development similar to *dsxB* in *Z. cesonia* that also lacks OD2. In termites, there appears to have been an ancestral loss of the *dsx* OD2 associated with evolution of eusociality (Miyazaki et al 2021). In these termites, instead of sex-specific alternative splicing, *dsx* appears to only be expressed in males. These systems offer a comparative approach to studying how alternative isoforms versus gene duplications that lack a protein-binding domain may impact the regulatory evolution of sexual dimorphism.

### An Important Role of MTs in Scale Cytoskeletal Structures and Growth

Like bristles on *Drosophila* wings, butterfly scales can be an excellent model system to study the role of the cytoskeleton during polarized cell growth (Wulfkuhle et al. 1998; Bitan et al. 2012; Dinwiddie et al. 2014). In *Drosophila* bristle development, MTs densities are high on the leading tip, which we also observed in the *Z. cesonia* wing scales. We also see MTs distributed laterally, parallel to where chitinous ridges develop on the upper surface. These ridges are the structures responsible for UV reflection, which are thought to “self-assemble” as chitin is deposited in pulses on the scale surface (Ghiradella 1994). In *Drosophila* bristles, MTs are important for trafficking vesicles of construction materials needed for chitin metabolism (Bitan et al. 2010). In butterfly scales, actin filaments and MTs may provide the tracks for the transport of materials from cellular organelles to sites of chitin deposition. The change in MTs in *Z. cesonia* scales from 48 to 72 h APF in the UV-reflecting region of male wings may be involved in regulating the pulsed deposition of chitin that generates the UV-reflecting structures. Interestingly on female wings, MTs were densely distributed and showed little differences across the wing, which corresponds with females lacking the UV-reflecting structures and in general have less differentiation in scale structure. An important role of MTs in scale biogenesis is also supported in Monarch butterflies, where a MT-related gene was proposed to underlie a depigmented state in a naturally occurring mutant (Kronforst 2015). Further investigations of MTs in butterfly scales may be a promising approach to better understanding the cellular processes that regulate variation in cell growth.

### Shared Genetic Mechanisms of Dosage Compensation and Sex-Specific Expression in Lepidoptera

In the silk moth, *Bombyx mori*, sex-specific splicing of *dsx* and dosage compensation appear to be controlled by a shared suite of genes, *BmMasc*, *BmPSI*, *BmIMP*, and *BmZnf2* (Gopinath et al. 2016; Katsuma et al. 2019; Kiuchi et al. 2019). The masculinization gene, *BmMasc*, is regulated by a piwi-interacting RNA that originates from the W chromosome, and acts as the genetic sex determination switch (Kiuchi et al. 2019). When *BmMasc* is inhibited, it impacts dosage compensation and sex-specific splicing of *dsx* (Kiuchi et al. 2019). *BmZnf2* appears to interact with *BmMasc*, *BmPSI*, *and BmIMP*, and when inhibited impacts both dosage compensation and *dsx* splicing (Gopinath et al. 2016). Interestingly, in *B. mori, dsx^F^* isoforms include all exons, and it’s only *dsx^M^* that experiences exon skipping when spliced. Therefore, there is no special alternative splicing machinery needed for *dsx^F^* to be transcribed, and when *BmMasc* or *BmZnf2* are inhibited, *dsx^F^* isoforms are still produced (Gopinath et al. 2016; Kiuchi et al. 2019). We were able to identify homologs for *BmMasc*, *BmPSI*, *BmIMP*, and *BmZnf2* in the *Z. cesonia* genome (XP_037869585, XP_012545114, XP_021206051, XP_004924549). However, we were only able to detect very low levels of expression for *BmPSI*, *BmIMP*, and *BmZnf2*, and we found no RNA-seq reads that mapped to *BmMasc*. This perhaps is not surprising if they are only involved in *dsx^M^* alternative splicing, as *dsx* was very lowly expressed in male wings.

We did find clear evidence of incomplete dosage compensation that suggested expression of Z-linked genes is actively being down-regulated in males of *Z. cesonia*. Down-regulation of Z-linked genes in males is consistent with findings in *B. mori and Heliconius* butterflies (Walters and Hardcastle 2011; Walters et al. 2015). In *B. mori*, male down-regulation of the Z was confirmed through RNAi knockdown of *BmMasc*, which led to a global increase in expression of Z-linked genes (Kiuchi et al. 2019). Despite similar observations of Z chromosome down-regulation in *Z. cesonia* males, there was no evidence of *masc* expression, suggesting an alternative molecular mechanism is likely involved. Due to the lack of *masc* expression, the low level of *dsx^M^* expression, and lack of alternative splicing for female *dsx* isoforms in *Z. cesonia* wings, we are unable to address if the same genes may regulate both dosage compensation and *dsx* splicing, as has been found in *B. mori*. However, it would be very interesting to explore this in *Z. cesonia* with the incomplete dosage compensation and potential involvement of the novel *dsx* duplication.

### *Dsx* and the Evolution of Sexual Dimorphism

Ultimately, some of the most remarkable morphological variation of traits in nature lies in the differences between males and females. Our understanding of the genetic basis of sexually dimorphic traits in animals has been revolutionized in the past few decades with advances in our understanding of *Dmrt* genes (Yang et al. 2008; Kopp 2012). For example, studies of the iconic horns on rhinoceros beetles and the sex combs on fruit fly appendages have shown *dsx*, the insect homolog of *Dmrt1*, is essential for the development of these sexually dimorphic traits (Kijimoto et al. 2012; Ito et al. 2013; Morita et al. 2019; Rice et al. 2019). Our findings suggest the DNA-binding motif of *dsx* may be very similar in Lepidoptera and other insects, yet *dsx* may regulate sexually dimorphic development through novel molecular mechanisms in *Zerene*. The well-characterized alternative splicing of male and female isoforms (*dsx^M^* and *dsx^F^*) that drives sexual dimorphic development in insects appears to have been replaced in wing development by sexually dimorphic expression of recently evolved paralogs. These paralogs would be released from the sexually antagonistic mutations that can be difficult to overcome when exons are shared in alternative splicing, like the *dsx^M^* and *dsx^F^* isoforms found across most insects. This would allow for the rapid amino acid evolution we find between the *dsx* paralogs in *Z. cesonia*. In addition, it opens opportunity for rapid diversification of regulatory modules that could independently control sex-specific spatial expression of the *dsx* paralogs. This is very different from *Drosophila* and other insects, where genes such as *sex lethal and transformer* are required for the sex-specific alternative splicing, and *cis*-regulatory modules are responsible for tissue-specific expression patterns (Rice et al. 2019). We hypothesize that the duplication of *dsx* would promote the evolution of not only tissue-specific, but also sex-specific regulatory modules. Duplications of *Dmrt* genes have been reported in diverse organisms ranging from vertebrates (Ogita et al. 2020) to hexapods (Price et al. 2015; Panara et al. 2019; Gruzin et al. 2020) and may be a key step in the evolution of complex sexually dimorphic traits. In this regard, our findings of the *dsx* paralogs highlight *Z. cesonia* as a potentially powerful system to study the evolution of molecular mechanisms that regulate sexually dimorphic development.

## Materials and Methods

### Experimental Design

To characterize sex-specific patterns of gene expression, mRNA sequencing was performed across seven developmental stages (late fifth instar larvae, 24, 48, 72, 96, 120, 148 h APF) for replicate males and females (42 samples total; minimum three replicates per sex, for each stage). To identify sex-specific expression patterns involved in sexually dimorphic development of UV-reflecting scales, analyses were focused on 24–72 h APF when scales differentiate and the UV-reflecting structures develop (Ghiradella 1989; Dinwiddie et al. 2014). Copies of the *dsx* gene were further examined through detailed genomic annotations, in situ hybridizations and antibody immunofluorescence. Differential expression of MTs in developing scales in males and females was also examined using antibody immunofluorescence. Lastly, potential targets of *dsx* were identified by performing DNA-binding motif enrichment analyses among subsets of genes with sex-biased expression. Collectively, these approaches provide a detailed view of sex-specific patterns of gene expression during scale development, with particular focus on the involvement of *dsx*.

### Preparation for RNA Sequencing

Butterflies were reared in controlled conditions (Shelby et al. 2020), and wing discs were dissected and preserved in RNAlater. Dissection timing varied within 4 h among samples within the sampled developmental stages.

### Transcriptome Sequencing and Assemblies

RNA-seq libraries were prepared from dissected forewings from individual butterflies using Nextera XT DNA Library Preparation Kit (Illumina, San Diego, CA, USA) and sequenced at 150-bp paired ends reads (Fenner et al. 2020). RNA-seq reads were preprocessed using AfterQC (Chen et al. 2017) and Trimmomatic (Bolger et al. 2014).

Two transcriptome assemblies were generated de novo using the evidential gene (EG) pipeline (http://arthropods.eugenes.org/EvidentialGene/; last accessed April 3, 2020) and the Oyster River protocol (MacManes 2018). The quality of the resulting transcriptomes was estimated using QUAST scores (Gurevich et al. 2013), BUSCO completeness (Seppey et al. 2019), BLASTX (Altschul et al. 1990) hits against a reference lepidopteran protein library, and mapping quality support using Bowtie2 (Langmead and Salzberg 2012). The assembly with the best quality (EG) was manually curated, removing transcripts smaller than 500 bp and redundant transcripts using CD-HIT-EST (Li and Godzik 2006) with a similarity threshold of 95%. The final transcriptome was annotated using Trinnotate following the procedure in Bryant et al. (2017) using the Trinotate pipeline with the uniprot/uniref 3.1.1 database for protein sequences references, and the PFAM 3.1 database for protein domain references. Scripts and reference files are accessible at the GitHub repository for this project (https://github.com/LF-Rodriguez/Zerene_cesonia_WingDiscDevelopment; last access April 3, 2020).

### Transcriptome Analysis of Wing Development

RNA-seq reads were used to estimate gene expression during wing scale development and identify gene networks associated with specific stages of wing scale development. First, reads were mapped to a reference transcriptome using Bowtie2 and gene expression was estimated using RSEM (Li and Dewey 2011) with default parameters. The R package WGCNA (Langfelder and Horvath 2008) was used to perform gene network analysis and measure the associations with specific developmental stages. *Dsx* transcripts and reads were also mapped to the reference genome of *Z. cesonia* (Rodriguez-Caro et al. 2020) to manually annotate *dsx* paralogs and examine read coverage of *dsx* exons.

### Analyses of Sexually Dimorphic Expression

Differences in scale morphology between sexes are evident by 72 h post pupation. We performed differential expression tests between sexes with the RNA-seq data sampled from at three stages of scale development: 24, 48, and 72 h APF. Differential expression analyses were performed using the R package DESeq2 (Love et al. 2014). Transcripts were considered DE between sexes with a *P*-adjusted value lower than 0.05 and log2-fold change higher than 2. Genes with male-biased and female-biased expression were identified as those that were significantly DE between sexes and higher in males or females, respectively. The R package TopGO (Rahnenfuhrer 2019) was used to test for GO enrichment on the set of DE genes using a customized GO annotation.

To test for dosage compensation on the Z chromosome during wing development, gene expression levels were compared between autosomal genes and Z-linked genes separately in males and females using normalized read counts (FPKM). For this analysis, lowly expressed genes (FPKM < 1) were removed, as well as genes that showed sex-biased expression during wing development following Huylmans et al. (2017). Differences in median expression between autosomes and Z chromosomes were detected using a *t*-test, and differences in gene expression levels in Z-linked genes between males and females were detected using a Wilcoxon test.

### Tissue-Specific Expression of *Dsx*

To examine *dsxA and dsxB* expression in tissues besides pupal wings, we used cDNA sequencing and explored available RNA-seq datasets. We dissected the following tissues and extracted RNA for 3–6 males and females: adult head, adult thorax, adult abdomen, adult gonads and fifth instar larval gonadal masses. mRNA was extracted with a Qiagen Total RNA isolation kit, and cDNA was synthesized using random primers and reverse transcriptase. Primers were designed from *dsxA* tand *dsxB* transcript and reference genome sequence alignments (*dsxA* exon1: P5-ATAGAGCTCAAGGGCCACAA, P2-GGGACCGAATCCACTGAACT; *dsxB* exon1: P5-ATAGAGCTCAAGGGCCACAA, P8-AAGCCGAATCCACTGAACTG; *dsxA* exons2-6: F3-TCGAGAACTGTCACAAGCTG, R2-AAACAGGCGTAACGAAGTTG). PCR products were amplified, cloned, and sequenced. Sequences were aligned to *dsxA* and *dsxB* transcript and reference genome sequence to characterize *dsx* isoforms present in each sample. We also used available RNA-seq data from male head and thorax previously used to annotate the reference genome (Rodriguez-Caro et al 2020), which we mapped to the reference transcriptome to determine isoform composition. To determine which *dsx* paralog was present in each RNA-seq and cDNA sequence, we used the substitution differences between *dsxA* exon 1 and *dsxB* shown in supplementary figure S3, Supplementary Material online. In addition, as a positive control to confirm the PCR primers could successfully amplify both *dsxA and dsxB*, we conducted PCRs using cDNA from a subset of male and female pupal wing RNA-seq samples that we confirmed had *dsxA and dsxB* transcripts present, respectively. These positive controls allowed us to infer that a lack of *dsxB* sequences in any tissue samples reflected a lack of *dsxB* transcripts, rather than an inability to PCR amplify *dsxB*.

### *Dsx* Annotation and Evolutionary Analyses

To annotate exon boundaries in *dsx* transcripts, we aligned each of our *dsx* transcripts to reference *dsx* exons from *Bombyx mori* (Zheng et al. 2019) using MAFFT (Katoh et al. 2002). We then mapped each *dsx* exon to the genome assembly of *Z. cesonia* (Rodriguez-Caro et al. 2020) using Gmap (Wu and Watanabe 2005). MUMmer (Marçais et al. 2018) was used to align the scaffold containing *dsx* against itself to identify duplications and estimate sequence conservation in the duplicates. TEs in the *dsx* gene were identified using RepeatMasker (http://www.repeatmasker.org). The NLS was identified in *dsxB* using NLSdb (https://rostlab.org/services/nlsdb/) that compares query sequences to a database of known NLS sequences. *Zerene eurydice dsxB* amplicons and sequences were obtained using the primers (FP-TGCATTGTGCACAGCAATAA and RP-AGTTCGGCGCTAGTTTGTGT). *Dsx* exon sequences for *Pieris rapae* (accession GCA_001856805.1) were downloaded from NCBI and *Colias eurytheme* sequences were acquired from a draft genome assembly provided by A. Martin and Chris Wheat. *Dsx* exon1 alignments were generated using the Muscle alignment tool and maximum-likelihood phylogenies were generated using a Tamura-Nei model with uniform rates and 1,000 replicate bootstraps to assess support, as implemented in MegaX Version 10.1.8 (Kumar et al. 2018). Trees were created from Newick files using the Ape package in R (Paradis 2012). Divergence time estimates for the *Colias/Zerene and Z. cesonia/Z. eurydice* lineage splits were taken from Zhang et al. (2019). Using Zhang et al. (2019) divergence time estimates for the *Colias/Zerene* (28.85 My) and *Z. cesonia/Z. eurydice* (1.76 My) lineage splits, and calibrated branch lengths from the nuclear genome tree of Zhang et al. (2019), we estimated a *Colias-Zerene* nuclear substitution rate of 0.0046 substitutions/my. We used this substitution rate and the number of substitutions between *Z. cesonia dsxA and dsxB* to estimate the divergence time for the paralogs (e.g., age of duplication event).

### *Dsx*-Binding Site Analyses

mRNA transcripts that correspond to genes exhibiting significant male-biased expression on 72 h APF were used to test for enrichment of the *dsx* DNA-binding motif. Aligned genomic intervals were used to extract 2 kb of sequence up- and downstream to use as input. These extracted sequences were tested for enrichment of the *Drosophila melanogaster dsx* DNA-binding motif position weight matrix (Clough et al. 2014) using AME (https://meme-suite.org/meme/tools/ame) from MEME Suite 5.3.1, via the available web server. Fisher’s exact test was conducted alongside a set of shuffled input sequences with preserved sequence size of 1 bp. The same method was applied to 72 h APF female-biased transcripts.

### Immunostains and In Situ Hybridizations

To corroborate our findings from DE tests, and characterize the spatial expression patterns of MTs, we immunostained developing pupal wings with the Primary Monoclonal Anti-α-Tubulin antibody (catalogue #T6074, Sigma Aldrich) (1:500 dilution) and Alexa 488 Anti-Mouse IgG2a (γ2a) secondary antibodies (catalogue #SAB4600239, Sigma Aldrich) (1:1000 dilution) for male and female forewings. Doublesex immunostains were performed using primary antibodies DsxDBD (DSHB) at a dilution of 1:50, and Alexa 488 Goat anti-mouse secondary antibody (ThermoFisher #A28175, 1:250 dilution). The time of pupation was recorded, and wings were dissected at 48, 72, 96 and 120 h post pupation, with a minimum of five replicates for each sex and stage. Dissection timing varied within less than 2 h across samples within the sampled developmental stages. Nuclear counterstaining were performed by mounting wings in a solution of Fluoroshield with DAPI (catalogue #F6057, Sigma Aldrich). A Leica SPE confocal microscope was used to collect fluorescent images of antibody stains.

We focused on characterizing spatiotemporal expression patterns of *dsx* transcripts and isoforms in developing wings using in situ hybridization. A fragment of *dsx* mRNA was amplified from *Z. cesonia* cDNA using primers specific for *dsxA* transcripts (FP-GTGCCTCCAGCTCCGC, RP-GTTCAGGATCACGAGCACGA). The amplicon with T7/SP6 overhangs were used as a template to synthesize DIG-labeled RNA probes. Fore and hind wing discs were collected from male and females at 72 h APF and used for in situ hybridization using a preestablished protocol (Martin and Reed 2014).

### CRISPR/Cas9 Editing

CRISPR/cas9 injections for *dsx* knockouts were attempted on a total of 450 individuals but resulted in lethality of all injected individuals before the fourth larval instar. Two *dsx* single-guide RNAs, *dsx1*: CCCGGTGTATCGTAAATGATAGT and *dsx2*: CCAACATATGATCCACTTTATTG, were designed and purchased from Integrated DNA Technologies as Alt-R crRNA in the Alt-R CRISPR-cas9 system. Embryo injections used borosilicate glass needles, and eggs were affixed to glass slides with double-sided sticky tape. Injections occurred between 3 and 5 h after eggs were laid. Fluorescein dye at 2.5 mg/ml was added to the injection mixture to ensure embryos were equally and evenly injected. After injections, eggs were kept in a petri dish in a rearing chamber with 50% humidity, 16:8 h photoperiod, and 27 °C temperature. After 3 days, host plant cuttings were laid across the slide, allowing larvae to immediately climb onto host plants once hatching occurred on day four of embryo development. Larvae were reared on an unlimited supply of host plant *Dalea purpurea*. Two experiment concentrations of cas9 and guide RNAs were used among the 450 individuals: 187 eggs were injected with 1 µg/µl cas9 and 222 ng/µl guide RNAs with a hatching success rate of 14% and 263 eggs were injected with 0.5 μg/µl cas9 and 111 ng/µl guide RNAs with a hatching success rate of 17%. Larval growth time was increased by more than double normal developmental times (Shelby et al. 2020) and all larvae died before reaching the fourth larval instar.

## Supplementary Material

Supplementary data are available at *Molecular Biology and Evolution* online.

## Supplementary Material

msab228_Supplementary_Data
