# Supplementary material for: Novel Doublesex Duplication Associated with Sexually Dimorphic Development of Dogface Butterfly Wings
Source: Mol Biol Evol. 2021 Jul 29;38(11):5021–33. doi: 10.1093/molbev/msab228 (PMC8557438; doi:10.1093/molbev/msab228)
Supplement: msab228_Supplementary_Data [file msab228_supplementary_data.pdf]

Supplementary Materials for

Novel doublesex duplication associated with sexually dimorphic development of dogface butterfly wings

Fernando Rodriguez-Caro,* Jennifer Fenner, Shivam Bhardwaj, Jared Cole, Caleb Benson, Alexandra M. Colombara, Riccardo Papa, Matthew W. Brown, Arnaud Martin, Ryan C. Range, Brian A. Counterman*

* Corresponding Authors, luis.rodriguezcaro@umontana.edu; bac0071@auburn.edu

This PDF file includes:

- Supplementary Text
- Figs. S1 to S9
- Table S1

Supplementary Text

RNA sequence library preparation

RNA was obtained using a guanidinium thiocyanate-phenol-chloroform extraction method with TRizol Reagent (TIANGEN, Beijing, China). Total RNA was eluted with 50µl of nuclease-free H2O and the RNA quantity was determined on a Qubit 2.0 fluorometer (Thermo Fisher Scientific, Carlsbad, CA, USA). To assess the quality of total RNA, 5 ul total RNA were added to 10 ul of molecular biology grade formamide (Fisher Scientific, Pittsburgh, PA) and incubated at 65 C for 10 min then at 4 C for 3 min to relax secondary structures of the RNA. Then the RNA was assessed through electrophoresis in 1.8x Tris-borate-EDTA (TBE) agarose gel. A total of 50 µl (roughly 120 ng/ul) of total RNA was used to isolate mRNA using oligo(dT) magnetic beads from NEBNext Poly(A) mRNA Magnetic Isolation Module (NEB, Ipswich, MA, USA). Full length cDNA was constructed from mRNA using a modification of the Smart-Seq2 method (Picelli et al. 2014). First, reverse transcription of isolated mRNA with Superscript IV reverse transcriptase (RT) (Thermo Fisher Scientific) and oligoDT primers (5′-AAGCAGTGGTATCAACGCAGAGTACT30VN-3’). When the RT reaches the 5’ end of the mRNA molecule the nontemplate-directed terminal transferase activity of the RT adds 2-5 Cs. These cysteines pair with the 3 riboguanosines at the 3’ end of a Linked Nucleic Acid containing template switching oligo (LNA-TSO: 5′-AAGCAGTGGTATCAACGCAGAGTACrGrG+G-3’) which permits the RT to switch templates and synthesize the complementary strand. At this point all single stranded full-length cDNA species contain known ends complementary to the below ISPCR primers with the same sequence on each end. Double stranded cDNA is synthesized with ISCPR primers (5′-AAGCAGTGGTATCAACGCAGAGT-3′) and KAPA HiFi HotStart Ready Mix (KAPA BioSystems). Approximately 0.2 ng/ul of resulting double-stranded cDNA was used to prepare cDNA libraries using Nextera XT DNA Library Preparation Kit (Illumina, San Diego, CA, USA) following the manufacturer’s instructions.

RNA-seq reads were preprocessed using AfterQC (Chen et al., 2017) and Trimmomatic (Bolger et al., 2014). AfterQC was used to correct base calls and remove poly-X tails in reads using a threshold of 12 bp. Trimmomatic was used to remove contaminants and low quality reads or read fragments as follows: First ILLUMINACLIP

was used to remove sequencing adapters. Next the leading and trailing ends of the read were trimmed with a quality threshold of 25. A sliding window of 10bp from the end of the read was then used, truncating the read when the average quality dropped below 25. After this, reads shorter than 36 bp were discarded. After filtering ~856.9 million reads, distributed across 36 samples, were kept for analysis (144 hrs APF samples were excluded from analysis due to low quality).

Supplemental Figures

Assembly	Sample	K-mer size	dsxB	dsxA
5M21K	Male day 5	21	0	1
5M25K	Male day 5	25	0	1
5M65K	Male day 5	65	0	1
5M75K	Male day 5	75	0	1
5M81K	Male day 5	81	0	1
TH65K	Male thx & head	65	0	1
TH75K	Male thx & head	75	0	1
TH81K	Male thx & head	81	0	1
1M21K	Male thx & head	21	0	1
6M25K	Male day 6	25	1	0
6M65K	Male day 6	65	1	0
3F21K	Female day 3	21	1	0
3F25K	Female day 3	25	1	0
3F63K	Female day 3	63	1	0
3F75K	Female day 3	75	1	0
3F81K	Female day 3	81	1	0
5F65K	Female day 5	65	1	0
6F63K	Female day 5	63	1	0
6F75K	Female day 6	75	1	0
6F81K	Female day 6	81	1	0
6F21K	Female day 6	21	1	0
4F25K	Female day 4	25	1	0
4F81K	Female day 4	81	1	0
4F65K	Female day 4	65	1	0
4F21K	Female day 4	21	1	0
4F75K	Female day 4	75	1	0

Figure S1. Independent transcriptome assemblies from individual samples, generate *dsxA* in male samples, and *dsxB* in female samples.

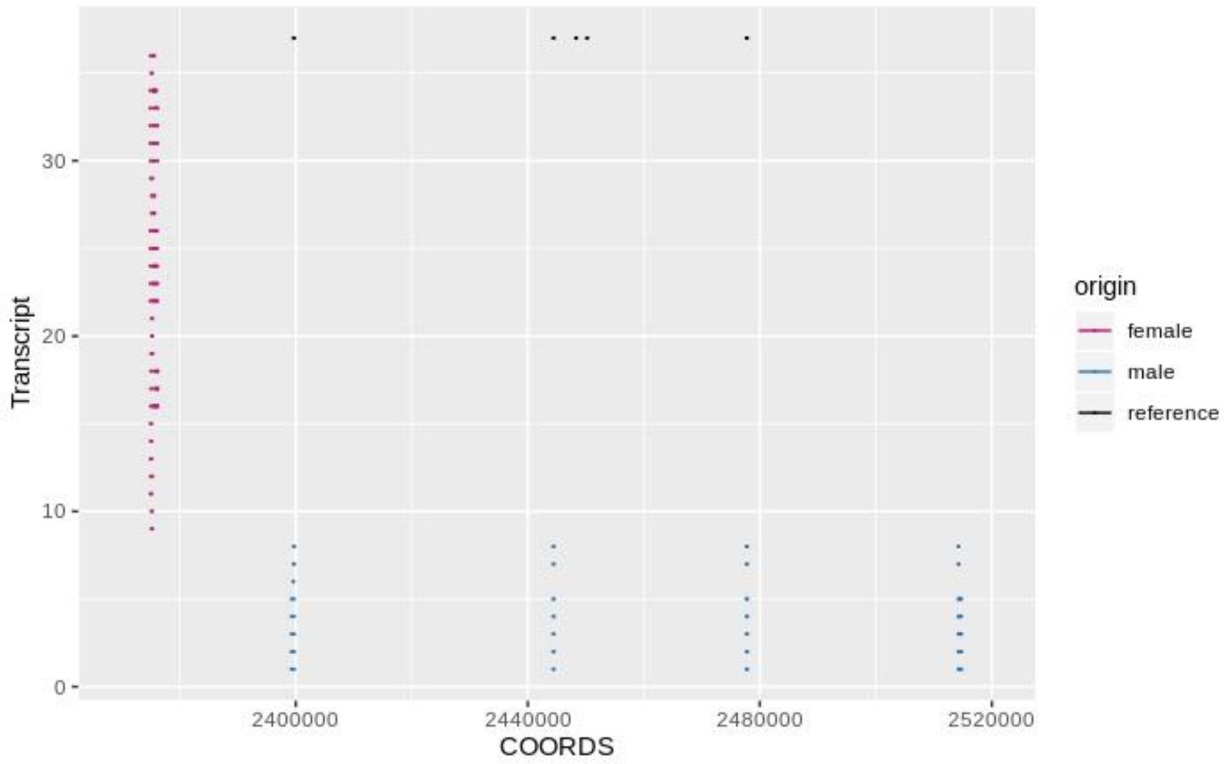

Figure S2. *Dsx* transcripts assembled from individual female samples and individual males samples mapped to reference *Z. cesonia* genome. All *dsx*^{F} transcripts mapped to *dsxB* and all *dsx*^{M} transcripts mapped to *dsxA* exons 1,2,5 and 6, as expected.

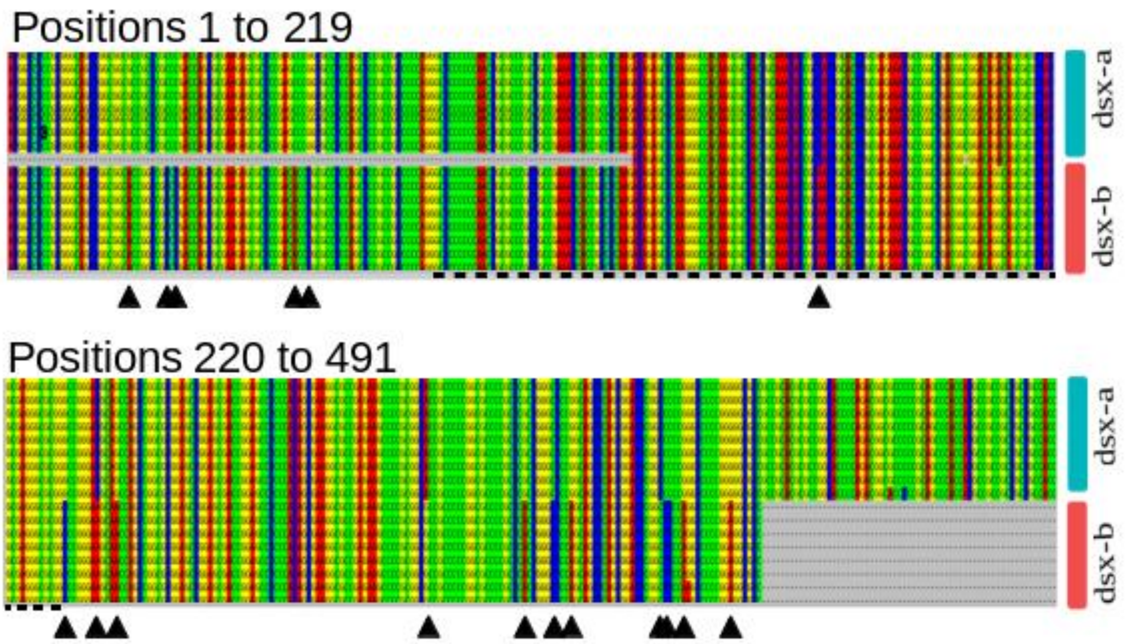

Figure S3. Alignment of *dsxB* transcripts to the coding sequence of *dsxA* exon 1. Triangles indicate polymorphisms between paralogs. Black dotted line below alignments denotes DM2, DNA binding domain.

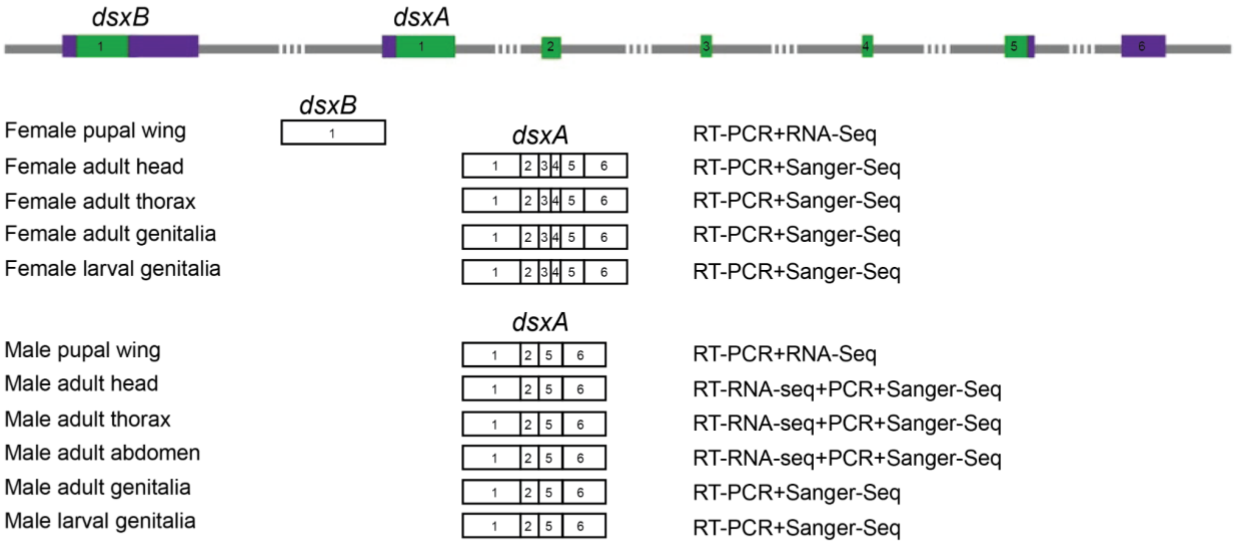

Figure S4. Sex and Tissue-specific *dsx* transcripts. Top shows annotation of *dsxB* and *dsxA*. The left column lists the tissues RNA was extracted from, and the center columns show graphic illustrations of the exons detected in the transcripts. The right column denotes the type of evidence used to confirm the transcript compositions in each tissue. Negative results for transcript detection are determined by a lack of *dsxB* sequences among Sanger sequences of cloned *dsxB* exon1 PCR products.

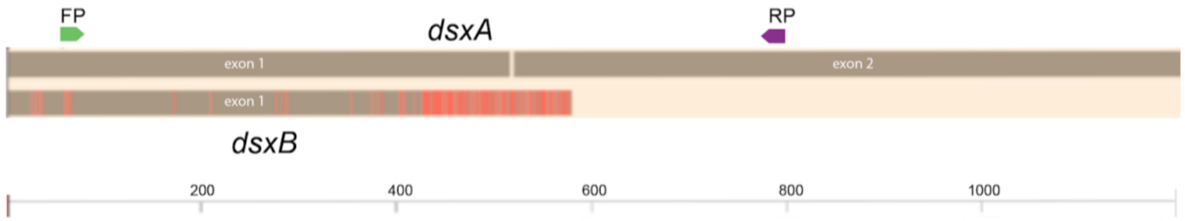

Figure S5. Alignment of *doublesex* transcripts and positions of the primer pairs used for riboprobes in dsxA *in situ* hybridization experiments (FP- Forward Primer, RP- Reverse Primer, red lines = polymorphic sites in alignment).

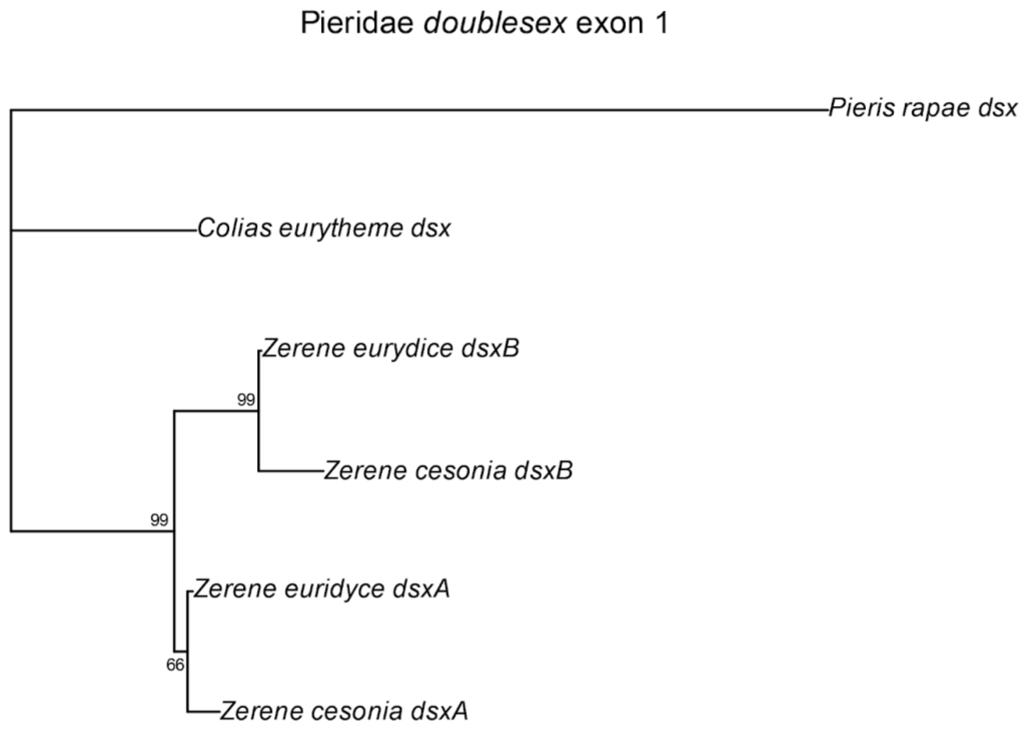

Figure S6. Maximum likelihood phylogeny of Pieridae *dsx* exon 1 sequences.

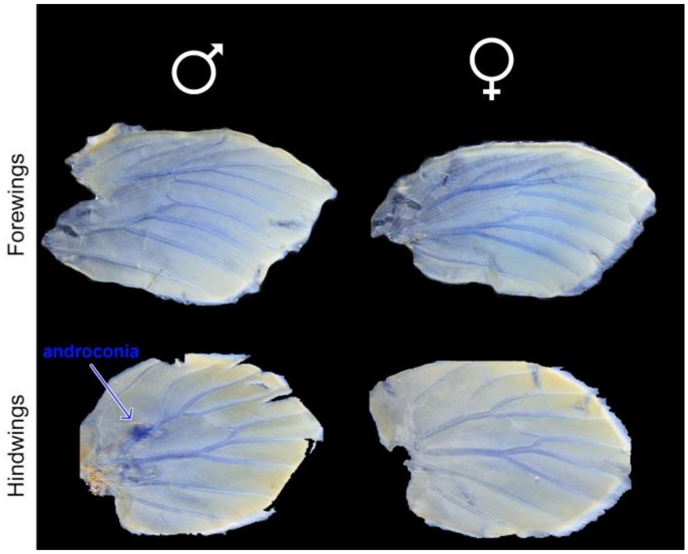

Figure S7. *In situ* hybridization shows *dsx* being expressed in male hindwing androconia at 48hrs APF.

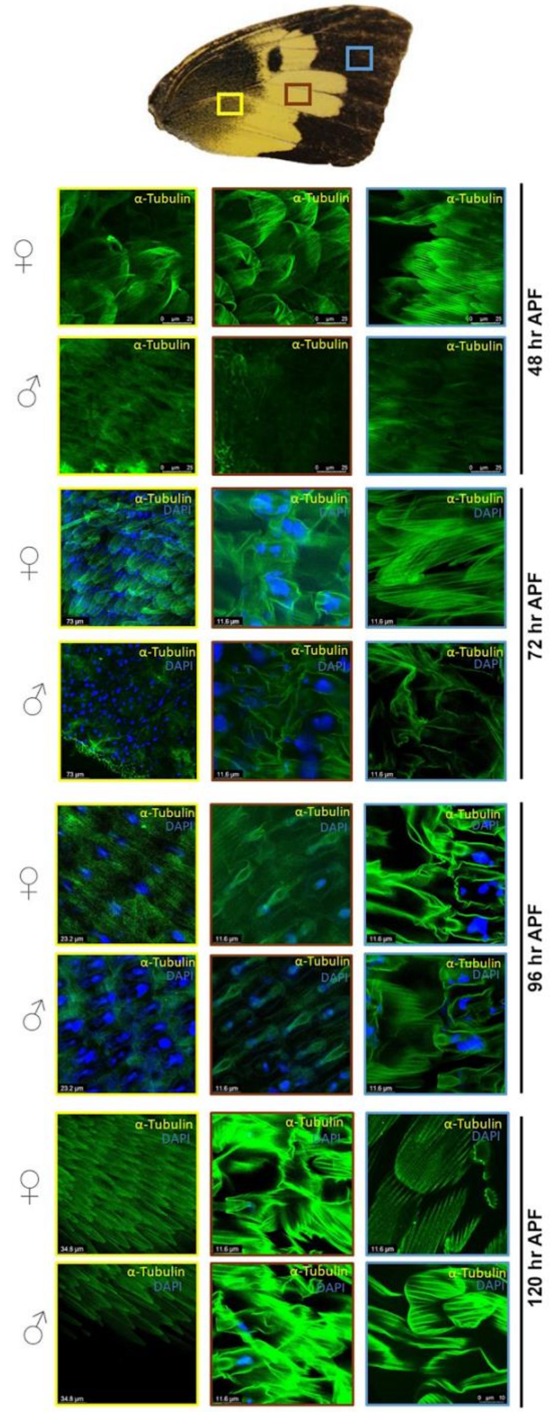

Figure S8. Microtubule distributions across wing regions (24, 48, 72, 96 and 120 hrs APF).

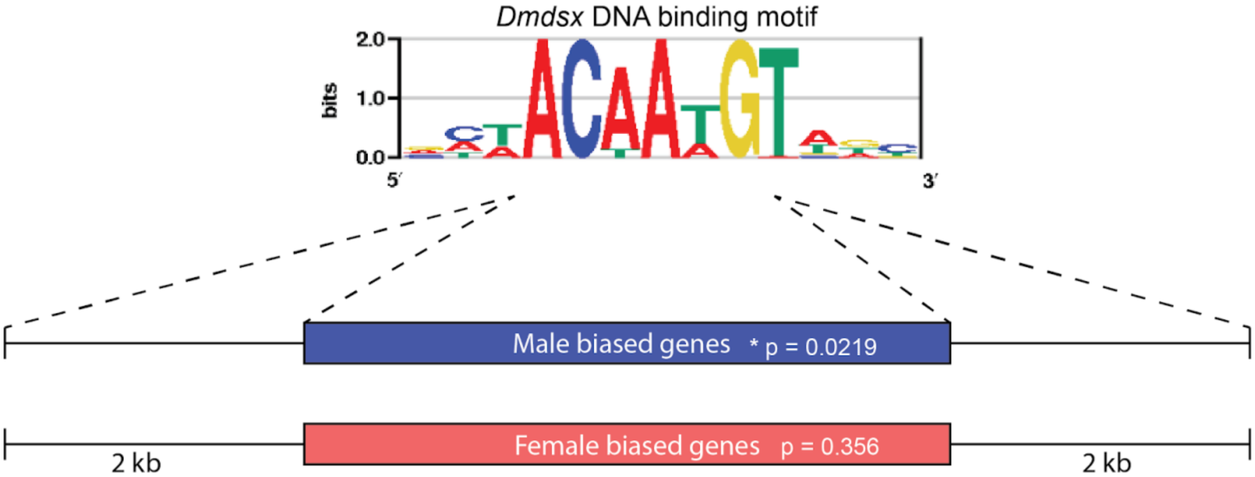

Figure S9. *Dsx* DNA binding motif enrichment in promoter regions of male, but not female biased genes at 72 hrs APF.

Supplemental Table

Sps 1	Sps 2	dN	dS	dN/dS
Z. ces_alpha	*Z. eur_alpha*	0	0.026	0.001
Z. ces_beta	*Z. eur_beta*	0.012	0.018	0.665
**Copy 1**	**Copy 2**			
Z. ces_alpha	*Z. ces_beta*	0.024	0.122	0.2
Z. eur_alpha	*Z. eur_beta*	0.012	0.057	0.209
**Sps 1**	**Sps 2/Copy1**			
C. eur	*Z. ces_alpha*	0	0.039	0.001
C. eur	*Z. eur_alpha*	0.006	0.337	0.019
**Sps 1**	**Sps 2/Copy2**			
C. eur	*Z. ces_beta*	0.029	1.008	0.029
C. eur	*Z. eur_beta*	0.017	0.908	0.019

Table S1. Pairwise non-synonymous and synonymous substitution rates among *Zerene* and *Colias dsx* sequences.
